# Impact of Soil-Applied Biopesticides on Yield and the Postharvest Quality of Strawberry Fruits in Southeast Texas

**DOI:** 10.3390/plants14081197

**Published:** 2025-04-11

**Authors:** Maryuri T. Nuñez de González, Peter A. Y. Ampim, Rahmat Attaie, Eric Obeng, Selamawit Woldesenbet, Adela Mora-Gutierrez, Russell Wallace, Yoonsung Jung

**Affiliations:** 1 Cooperative Agricultural Research Center, College of Agriculture, Food, and Natural Resources, Prairie View A&M University, Prairie View, TX 77446, USA; 2 Formerly of the Cooperative Agricultural Research Center, College of Agriculture, Food, and Natural Resources, Prairie View A&M University, Prairie View, TX 77446, USA; 3 Formerly of the Department of Horticultural Sciences, Texas A&M AgriLife Research and Extension, Lubbock, TX 79403, USA; 4 Statistical Consulting Center, Department of Statistics, Texas A&M University, College Station, TX 77843, USA

**Keywords:** organic pesticides, Camino Real strawberry, physicochemical properties, sugar content, color

## Abstract

The production of organic strawberries (*Fragaria* × *ananassa*) in Texas is becoming more popular because consumers prefer locally grown berries and are willing to pay premium prices. However, local climatic conditions pose a high risk for insect problems and fungal diseases; hence, effective pest and disease management strategies are needed. Developing effective and safe methods of producing organic strawberries is necessary for meeting local consumer demand. Therefore, the objective of this study was to evaluate the impacts of selected commercially available soil-applied biopesticides on yield and the quality of Camino Real strawberries established using bare roots on plastic mulch-covered beds with drip irrigation. The ten biopesticide treatments were replicated three times in a completely randomized design. The berries used for this study were subsampled from harvests made in mid-April, late April, and mid-May 2019. Yield, biometrical characteristics, and physicochemical analyses such as pH, acidity, total soluble solids, sugars and organic acid contents, firmness, and instrumental color were determined for fresh strawberries after each harvest period. Experimental data were analyzed using the PROC Mixed model procedure. The effects of the soil-applied biopesticide treatments on strawberry yield varied. The results of strawberry yield suggest that biopesticides applied at the right time and frequency have the potential to perform at similar levels to their conventional counterparts. Camino Real strawberries treated with biopesticides, harvested during mid-April, late April, or mid-May, exhibited acceptable flavor based on the recommended values of TA and TSS for strawberries. The biopesticides showed no negative effects on yield and fruit quality and thus they could serve as alternatives to conventional products used.

## 1. Introduction

Consumer awareness of the impact of fruit intake on overall health and well-being is continually increasing. Therefore, the consumption of berry fruits such as strawberries (*Fragaria* × *ananassa*) has been promoted for their significant health benefits such as antioxidant, anti-inflammatory, and anti-diabetic activities [1,2]. Strawberry fruit is a valuable horticultural crop widely consumed due to its appearance, firmness, and chemical composition. These attributes determine the crop’s value and consumer acceptance of the fruit [3]. However, strawberry production exhibits enormous difficulties due to its susceptibility to pests and diseases. In the USA, the two top strawberry producers are California and Florida. However, strawberry production is increasing in Texas and some mid-southern states because of consumer interest in locally grown berries. Texas strawberry production is currently estimated at 350 to 400 acres. This production level represents approximately a 300% increase from 2010 [4]. The warm climate in the mid-south also increases the risk of strawberry disease and insect pests [5].

As a result, strawberry farmers often use conventional pesticides to protect crops from diseases (*Botrytis cinerea*, *Oidium fragariae*, and *Mycospharella fragariae*) and pests (*Steneotarsonemus fragariae*, *Anthonomus rubi*, and *Tetranychus urticae*) [6]. Thus, efficient protection is critical for the quality and yield of this commodity crop.

Conventional strawberry production has the potential to leave pesticide residue on the fruits. However, organic cultivation approaches minimize the negative impact of pesticide residues and hence are increasingly becoming more popular. Organic products have a comparatively lower potential for leaving residues on fruits sold in the market [7]. Using conventional pesticides such as fungicides can modify the chemical composition of the fruits and contribute to the risk of the residue contamination of the strawberry fruits [8]. This concern provides an opportunity for the use of biopesticides to control fruit diseases and pests instead of conventional pesticides.

According to the United States Environmental Protection Agency (EPA), biopesticides are usually inherently less toxic and effective in small quantities. Moreover, they often decompose rapidly which results in lower exposure thereby, avoiding the pollution problems caused by conventional pesticides. These biopesticides are derived from natural sources such as animals, plants, bacteria, and certain minerals [9]. The rapid growth of organic farming is due to consumer demand for healthier foods and government initiatives supporting the environmental sustainability of agricultural practices [10].

Normally, high-quality strawberries are selected based on several attributes, including color, shape, size, appearance, firmness, and flavor [11]. However, factors such as environmental conditions, agricultural practices, and harvest timing can significantly impact the quality of strawberry fruits [12]. In this study, we evaluated the impacts of selected commercially available biocontrol and organic products applied in a soil system on yield and the quality characteristics of Camino Real strawberries grown in southwest Texas.

## 2. Results and Discussion

### 2.1. Strawberry Yields

The effects of the soil-applied biopesticide treatments on strawberry (*Fragaria* × *ananassa*) yield varied (Table 1). Application frequency resulted in a significant difference for Regalia (T8 vs. T4) and Double Nickel (T9 vs. T5) in terms of marketable fruit numbers and weights (Table 2). Making three applications, one at transplanting and two subsequently at 8-week intervals, significantly increased (*p* < 0.05) fruit numbers and yield weights for Regalia- and Double Nickel-treated plants compared to the same treatments made in six applications, one at transplanting time followed by five additional treatments at 4-week intervals afterwards. Similarly, an increase of 36.7% and 49.6% was observed for fruit numbers and weights for Actinovate treatments for the same application frequencies (T6 vs. T10), but the difference was not statistically significant (*p* > 0.05). The reason for this is unclear. The opposite was observed for RootShield Plus treatments, where the six-time application treatment (T3) yielded 42.3% and 56.4% more fruit numbers and weights, respectively, compared to the three-time application treatment (T2). Strawberry yield (fruit numbers and weights) for Regalia (T8), Double Nickel (T9), and Actinovate (T10) applied in three applications were statistically similar to the standard conventional treatment (Ridomil Gold + Abound FL), which can also be considered as a positive control. Yields for these treatments were also significantly different from the untreated control (UCtrl, T1) as expected. However, the RootShield Plus treatments were mostly similar statistically to the untreated control. These results suggest that biopesticides applied at the right time and frequency have the potential to perform at similar levels as their conventional counterparts. These results are contrary to observations by Pruitt [13], which indicated no clear yield advantages for Camino Real and Sweet Sensation using biopesticides.

### 2.2. Biometrical Characteristics

The fruit length and major diameter were not affected (*p* > 0.05) by the biopesticide treatments in the strawberries harvested in mid-April or mild-May (Table 3). However, an increase in fruit length was noted in the Actinovate treatment applied in six applications (T6) compared to the untreated control (T1) when the strawberries were harvested in late April.

The length and major diameter of strawberry fruit are not commonly reported in the literature. In our study, the length and major diameter of tested fruits were similar to those values reported by several authors [3,11,14]. In contrast, a lower fruit diameter for the cv. Camino Real was reported by Chaves et al. [15]. Also, Muzzaffar et al. [16] observed low average fruit length and width in fresh strawberries (cv. Chandler). Overall, the Camino Real strawberries collected in our trial exhibited good biometrical characteristics in all the biopesticide treatments harvested during any period.

### 2.3. Total Soluble Solid Content, pH, and Titratable Acidity

The soil-applied biopesticide treatments had a significant effect (*p* < 0.05) on the total solid soluble (TSS) content, pH, titratable acidity (TA), and TSS/TA ratio in the Camino Real strawberries harvested at different periods (Table 3 and Table 4). Samples resulting from RootShield Plus applied in six applications (T3) that were harvested in mid-April presented greater TSS than the untreated control samples (T1). However, no significant differences (*p* > 0.05) were observed among the UCtrl, RootShield Plus applied in three applications (T2), RootShield Plus applied in six applications (T3), Regalia applied in six applications (T4), Double Nickel applied in six applications (T5), Actinovate applied in six applications (T6), Ridomil Gold + Abound FL applied in three applications (T7, positive control, +Ctrl), Double Nickel applied in three applications (T9), and Actinovate applied in three applications (T10) samples (Table 3).

Samples where RootShield Plus was applied in three applications (T2) that were harvested in late April exhibited the highest TSS as compared to the +Ctrl samples. On the other hand, samples where RootShield Plus was applied in six applications (T3) presented a lower TSS in Camino Real strawberries harvested during mid-May when compared to the +Ctrl and UCtrl samples (Table 3). Camargo et al. [17] reported that the cultivar Camino Real produced fruits with higher TSS (7.96 °Brix) in a conventional system. In contrast, Chaves et al. [15] and Zahid et al. [18] indicated low TSS of 5.1 ± 0.40 and 4.93 ± 0.76 °Brix, respectively, in the same cultivar.

The TSS consists of polysaccharides and organic acids found in fruits, impacting their flavor and indicating ripeness [19].

In comparison with the +Ctrl samples, lower pH was observed in the fruits of all treatments in the fruits harvested in mid-April, except in the Actinovate + 6A (T6) and Regalia + 3A (T8) (Table 4). The Regalia + 3A (T4) treatment exhibited the highest pH (3.83 ± 0.032) in the fruits harvested in late April. In the Camino Real strawberries harvested during mid-May, no significant differences (*p* > 0.05) were noted in the pH among treatment samples and the +Ctrl samples (Table 5). Pelayo-Zaldívar et al. [20] reported pH values of 3.6 ± 0.03, 3.7 ± 0.03, and 3.8 ± 0.03 in the strawberry cultivars Aroma, Diamante, and Selva, respectively, harvested in May. However, a higher pH (4.09 ± 0.14) has been reported in strawberry cv. Chandler [18]. The pH is an important indicator of fruit properties such as sourness and flavor [21]. The pH value increases as the fruit ripens and oxidizes over time [19].

In this study, the ranges of the TA (% citric acid) were 0.65–0.79%, 0.58–0.88%, and 0.64–1.24% in Camino Real strawberries harvested in mid-April, late April, and mid-May, respectively (Table 5). Camargo et al. [17] determined the acidity of berries from Camino Real was 0.94% in the conventional system. A percentage of TA of 0.84 had been reported in the Chandler cultivar by Zahid et al. [18]. However, Pelayo-Zaldivar et al. [20] reported TA values of 0.74, 0.87, and 0.75% in the cultivars Aroma, Diamante, and Selva, respectively, when the fruits were harvested in May. Depending on the cultivar and preharvest factors, TSS and TA in strawberries harvested at commercial ripeness varied from 5 to 12% and from 0.50 to 1.87%, respectively [20]. For an acceptable strawberry flavor, it is recommended to have a maximum of 0.8% TA and/or a minimum of 7% TSS [22]. Overall, the Camino Real strawberries treated with the biopesticides, harvested during mid-April, late April, or mid-May, exhibited acceptable flavor based on these recommended values of TA and TSS.

The TSS/TA ratio values ranged from 9.61 to 11.47 in Camino Real strawberries harvested in mid-April (Table 4). The highest TSS/TA ratio was observed for the Actinovate + 3A treatment (T10) compared to the +Ctrl in fruits harvested in mid-April. A lower TSS/TA ratio was observed for the RootShield Plus + 3A treatment (T2) compared to the +Ctrl in Camino Real strawberries harvested in late April (Table 4). The results observed in this study were higher than those reported by others for Camino Real. Pinelli et al. [23] and Chiomento et al. [24] indicated TSS/TA ratio values of 8.4 ± 0.2 and 4.55 ± 1.04, respectively. The relationship between TSS and TA is crucial for assessing fruit quality. The flavor of the ripe strawberry is in part determined by the sugars and acids ratio [23,25]. The TSS/TA ratio, as a measure of sweetness, is a good indicator of organoleptic evaluation for strawberries [25]. Likewise, in this study, we observed an acceptable relationship between sugar and acidity, TSS/TA ratio based on Pelayo-Zaldívar et al. [20].

### 2.4. Sugars and Organic Acids Contents in Strawberry

The soluble sugars, glucose, fructose, and sucrose were studied in Camino Real strawberry fruits. We found a significant difference (*p* < 0.05) in glucose and fructose contents in the harvested fruits when biopesticides were applied (Table 5). Compared to the +Ctrl or conventional pesticide, the highest glucose content was observed in strawberries harvested in mid-April from the Double Nickel + 3A treatment (T9) (Table 5). However, the highest fructose content was observed in strawberries from the Actinovate + 3A treatment (T10) harvested in mid-April (Table 5).

No significant differences (*p* > 0.05) were observed in the glucose content in the fruits harvested during late April when compared to the +Ctrl samples or conventional samples. Glucose concentrations were higher in RootShield Plus + 3A treatment (T2) samples harvested in mid-May compared to +Ctrl samples (Table 5). Schwieterman et al. [21] evaluated fresh strawberry (cv. Festival) harvested in winter and reported the contents of glucose, fructose, and sucrose were 1903, 2049, and 1218 mg/100 g in the early season 1 (week 2) and 1127, 1311, and 309 mg/100 g in the late season 1 (week 7). The authors noted that glucose, fructose, and sucrose represent the major soluble sugars present in strawberries [21]. The sucrose content in berries is lower than fructose and glucose contents [26]. In this study, we observed the same trends of glucose, fructose, and sucrose levels in the berries as compared to the reported values [21,26].

The total sugar, sweetness index, and total sweetness index were highest in strawberries from the Actinovate + 3A treatment (T10) harvested in mid-April compared to the +Ctrl samples. In the Camino Real strawberries harvested in mid-May, no significant differences were noted in the total sugar, sweetness, and total sweetness indexes when compared to the +Ctrl samples (Table 6). Total sugar contents of 5169 mg/100 g and 4490 mg/100 g have been reported in the cultivars Festival and Aromas, respectively [20,21]. An average total sugar content of 5250 ± 80.00 mg/100 g was observed by Muzzaffar et al. [16] in ripe strawberries (cv. Chandler).

One of the main parameters influencing the flavor of berries is the sugar content, which is considered an essential criterion for evaluating nutritive value and overall fruit quality. Sugar levels are affected by diverse factors including genetic factors, cultivation techniques, and preharvest conditions [27]. In general, the application of biopesticides in the soil did not adversely affect the soluble sugar or the total sugar contents of fresh strawberries in our experiment.

The results for organic acids contents (Table 7) demonstrate that the most abundant acid in Camino Real strawberries is citric acid which ranged from 0.58 ± 0.030 mg/100 g to 0.73 ± 0.030 mg/100 g in mid-April, 0.45 ± 0.017 mg/100 g to 0.69 ± 0.017 mg/100 g in late April, and 0.57 ± 0.029 mg/100 g to 0.70 ± 0.029 mg/100 g in mid-May. The highest (*p* < 0.05) citric acid contents in our trial were observed in samples from the Actinovate + 3A treatment (T10) and the RootShield Plus + 3A treatment (T2) harvested during mid-April and mid-May, respectively. We also noted higher (*p* < 0.05) malic acid contents in samples harvested in mid-April from the following treatments: Regalia + 6A (T4), Double Nickel + 6A (T5), Regalia + 3A (T8), Double Nickel + 3A (T9), and Actinovate + 3A (T10). No effects (*p* > 0.05) were observed on the malic acid contents of the Camino Real strawberries harvested in mid-May comparing the biopesticide treatments and the +Ctrl treatment (Table 7). Similar results for citric acid content were reported for Diamante (0.67 ± 0.03%) and Aromas (0.52 ± 0.03%) cultivars harvested in May by Pelayo-Zaldívar et al. [20]. However, these authors reported higher malic acid contents in Diamante (0.18 ± 0.02%) and Aromas (0.17 ± 0.02%) than those quantities observed in our study.

### 2.5. Firmness and Instrumental Color in Strawberry

Firmness is a major index for evaluating the quality of strawberry fruits and a desirable characteristic for consumers. Moreover, fruit firmness is associated with resistance to transportation and storage [3].

In our study, we did not observe any negative impact on the firmness of Camino Real strawberries following biopesticide treatments compared to the +Ctrl treatment (Table 7). Statistically higher (*p* < 0.05) values of firmness were noted in samples from the RootShield Plus + 3A treatment (T2), RootShield Plus + 6A treatment (T3), and Regalia + 6A treatment (T4) harvested in late April compared to the +Ctrl samples.

The biopesticides treatments also had a significant effect (*p* < 0.05) on the internal and external color parameters (Table 8 and Table 9). Compared to the +Ctrl samples, we observed a lower value of Chroma_i_ in samples from the Actinovate + 3A treatment (T10) harvested in late April (Table 8). However, the lowest values of Hue_i_ were noted in samples from the Regalia + 3A treatment (T8) and RootShield Plus + 3A treatment (T2) harvested in mid-April and mid-May, respectively, while the highest Hue_i_ value was observed in samples from the Double Nickel + 6A treatment (T5) harvested in late April (Table 8).

For the surface or external color parameters, lower values of Lightness_e_ were found in Camino Real strawberries from the RootShield Plus + 6A treatment (T3) and Regalia + 6A treatment (T4) harvested in mid-April compared to the +Ctrl samples (Table 9). Slightly darker colors were also noted in Camino Real strawberries from the Regalia + 3A treatment (T8) harvested in mid-May, indicating that the fruits in these treatments developed a slightly darker color. We also noted the lower value of Chroma_e_ and the highest value of Hue_e_ in the Double Nickel + 6A (T5) strawberries harvested during mid-April (Table 9). In fruits harvested in mid-May, we observed lower Chroma_e_ values for the RootShield Plus + 6A (T3) and Regalia + 3A (T8) treatments compared to the +Ctrl treatment (Table 9). We speculated that the effects observed for some of the biopesticide treatments could be associated with factors such as chemical composition (anthocyanins and sugar contents), harvest time, and application frequency. These results are different from those of Ornelas-Paz [3] who reported higher values of surface (external) color parameters L* (50.4 ± 1.1), Chroma (31.7 ± 1.1), and Hue (45.8 ± 2.6) in organic Albion strawberry cultivar (harvested with 75% red). These authors observed low Hue values (18.2 ± 0.6) in strawberries harvested that were dark red. The authors noted that this value fell into the reddest region of the chromaticity diagram, suggesting that the fruit in this stage was considerably rich in red pigments. Capocasa et al. [28] also reported higher L* (32.9 ± 0.4) and Chroma (42.0 ± 0.6) values on the color surface in the Camarosa cultivar than those observed in our study. In addition, Pelayo-Zaldívar et al. [20] reported high L* (33.2 ± 0.84) and Chroma (36.6 ± 1.05) values and low Hue (29.1 ± 1.13) values in the Aromas cultivar.

In general, the biocontrol and organic products in the soil did not adversely affect the internal and external color of Camino Real strawberry. The strawberry fruit color is an important attribute for consumer product acceptance and/or preferences.

Future research may include optimizing the application of biopesticides across various strawberry varieties and production systems. Additionally, evaluating the effects of these biopesticides on yield, quality, and nutritional values of postharvest strawberries will be essential for improving strawberry production practices.

## 3. Materials and Methods

### 3.1. Chemical and Reagents

The biopesticides used in this study were obtained from various commercial sources and are listed as organic inputs by the Organic Materials Review Institute (OMRI). However, the positive control treatment was used as a conventional standard for comparison purposes. Phenolphthalein, ethanol, and sodium hydroxide (NaOH) were purchased from Sigma-Aldrich (St. Louis, MO, USA). Orion buffer solutions (pH 4 and 7) were purchased from Thermo Fisher Scientific (Pittsburgh, PA, USA). Biochemical kits to quantify sucrose/D-glucose/D-fructose, citric acid, and L-malic acid (CAT# 10-716-260-035, CAT# 10-139-076-035, and CAT# 10-139-068-035) were obtained from R-Biopharm (Darmstadt, Germany). All chemicals were of analytical reagent grade and all solutions were prepared with deionized water that was obtained by passing distilled water over a mixed bed of a cation–anion exchange.

### 3.2. Soil Characteristics and Climatic Conditions of Study Site

The strawberry experiment was conducted at the Prairie View A&M University Research Farm (Longitude 30.080400, Latitude -95.990930) from October 2018 to May 2019 at a site that is not certified organic. The soil at the site is classified as sandy loam soil (fine-loamy, siliceous, semiactive, hyperthermic Oxyaquic Paleudalfs). The soil properties at the beginning of the experiment were as follows: pH 7.1, EC 1149 umho/cm, nitrate-N 200 mgL^−1^, phosphorus 195 mgL^−1^, potassium 312 mgL^−1^, calcium 2498 mgL^−1^, magnesium 174 mgL^−1^ and sulfur 157 mgL^−1^. Climatic conditions during the growing season are summarized in Table 10. Monthly precipitation for the growing season ranged from 14.98 mm (in October 2018) to highs of 250 mm (in May 2019). Seventeen precipitation events occurred in February 2019 and precipitation was the highest during the growing season. On the other hand, the highest temperature, relative humidity, and wind speed during the growing season were all recorded in May 2019 (Table 10).

### 3.3. Planting Material and Experimental Design

The variety Camino Real (*Fragaria* × *ananassa*) was planted on 19 October 2018, using bare roots. Twenty (20) bare roots were planted 0.3 m apart per plot on raised beds covered with black plastic mulch lined with drip tape for irrigation. The ten soil-applied biopesticides (Table 1) used were arranged in a completely randomized design with three replications per treatment. Timing of applications and rates used are also presented in Table 1. Water was used as the untreated control (UCtrl) to receive the same degree of wetness during pesticide application. Conventional pesticides (Ridomil Gold + Abound FL) were used as a positive control (+Ctrl). The crop received approximately 2.54 cm of water including rainfall per week. Fertilizer application was based on soil test recommendations. Nutrient requirements were met through fertigation using water soluble potassium nitrate (13-0-46). While weeds immediately around strawberry plants were pulled by hand, those between rows were managed through a tractor-driven rotary tiller.

### 3.4. Strawberry Harvesting and Sampling

**Figure 1:**
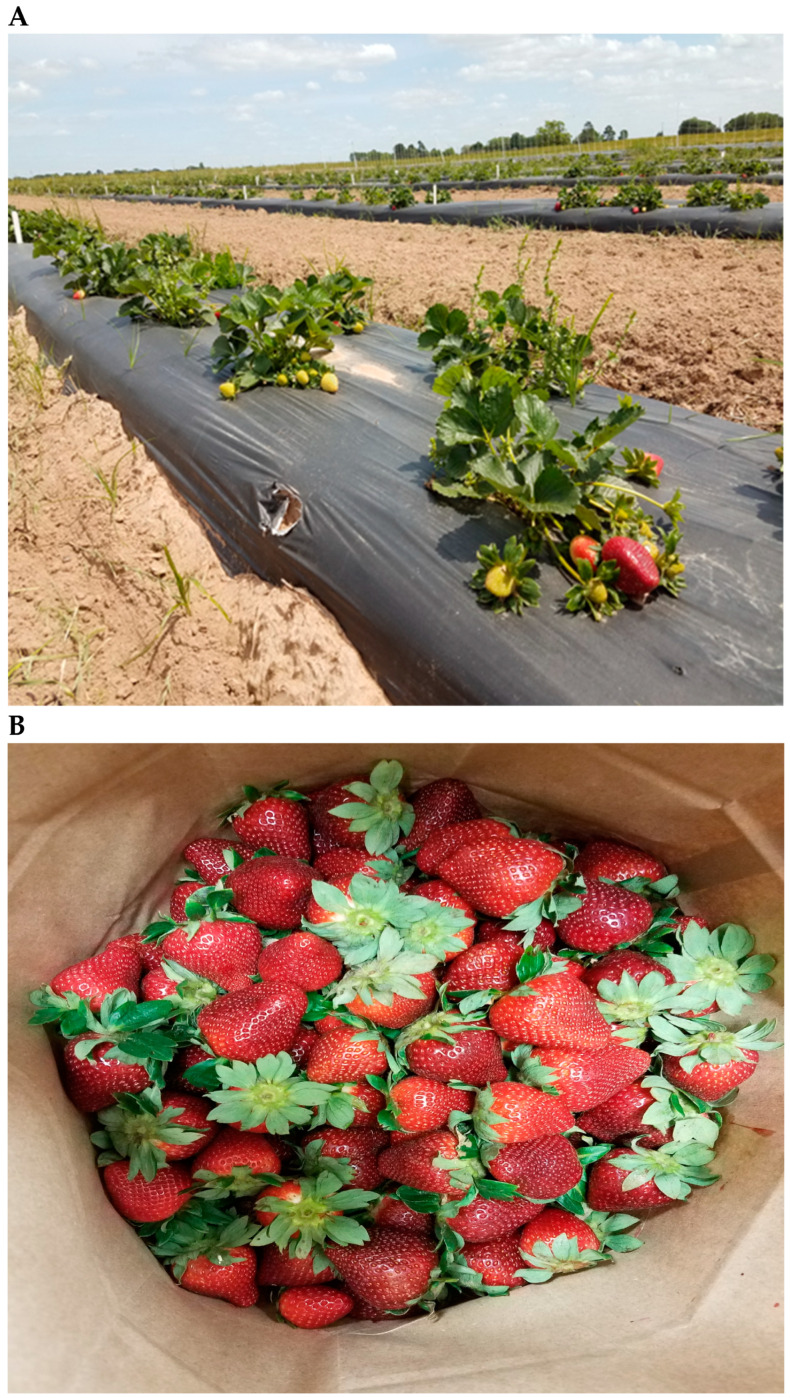
Photographs of Camino Real strawberry (*Fragaria* × *ananassa*) plants (**A**) and harvested fruits (**B**).

The strawberry fruits were harvested by hand when at least 75% ripe, every three days in the morning hours. During harvests, all strawberry plants (i.e., 20 plants/plot) with ripened fruits in each plot were harvested. Photographs of the strawberry plants and harvested fruits are shown in Figure 1A,B. The harvested fruits were sorted into marketable and culled categories, then counted and weighed. Strawberries used for the quality analyses were subsampled from marketable fruits. The strawberry fruits used for this study were harvested on April 16 (mid-April), April 30 (late April), and May 17 (mid-May) in 2019 and immediately transported to the laboratory while maintaining the cold chain (≤4 °C). The samples were visually inspected, and damaged strawberries were removed to ensure uniformity in color and size. Strawberry fruits (approximately 800 g of fruit) per treatment from each harvesting time were placed in refrigerated storage (4 °C) and subsampled (400 g) for fresh analysis within 1–3 days of receipt, except for the determination of biometrical characteristics, pH, titratable acidity (TA), total soluble solids (TSS), color (L*, a*, and b*), and firmness. Another set of subsampled strawberry fruits (400 g) were stored at –80 °C for later biochemical analysis such as sugars (glucose, fructose, and sucrose) and organic acids (citric and malic acids).

### 3.5. Determination of Biometrical Characteristics

Biometrical characteristics and visual shapes were immediately evaluated for 5 berry samples per treatment from each harvesting time. Fruits were washed and dried with paper towels, and the sepals were removed. The lengths and major diameters (mm) of the fruits were measured using a Mitutoyo digimatic caliper (Kiyohara, Japan).

### 3.6. Determination of pH, Titratable Acidity, and Total Soluble Solids

To determine pH, TA, and TSS of samples, three replicates of strawberry juices were used in this study. Strawberry samples were homogenized in a blender (Model WF2211314, Waring Laboratory Science, Torrington, CT, USA) to obtain the fruit juice. The TSS was determined by a direct reading at room temperature (~22 °C) using a refractometer (Model 300010; Sper Scientific Ltd., Scottsdale, AZ, USA). The refractometer was calibrated using deionized water and the results were reported in °Brix.

The TA in the fresh strawberry samples was determined according to the method of Perez Cayo et al. [29]. Briefly, the strawberry juice was centrifuged (Avanti J-E centrifuge, Beckman Coulter Inc., Indianapolis, IN, USA) at 17,800× *g* for 20 min. An aliquot (6 g) was diluted with 50 mL of deionized water. The mixture was titrated with standardized 0.1 N NaOH until the first color change, which signals the endpoint (at pH 8.1–8.3 endpoint), persisted for 30 s. Phenolphthalein (1% *w*/*v* in 95% ethanol) was used as an indicator. The results were reported in percentage of citric acid. The pH of the samples was measured using a benchtop pH meter (Accumet AE150, Fisher Scientific, Pittsburgh, PA, USA) at 25 °C. The pH meter was calibrated with reference buffers (4.00 and 7.00; Orion buffer solutions; Thermo Fisher Scientific, Pittsburgh, PA, USA). The ratio of total soluble solids to titratable acidity (TSS/TA) was also calculated.

### 3.7. Determination of Color and Firmness

The firmness of randomly selected fruits was measured using a TA.XTPlus Texture Analyzer (Texture Technologies Corp., Hamilton, MA, USA) fitted with a 5 kg load cell and controlled by a computer. The whole strawberries were cut in half to improve how they packed into the 13-tine Multiple Puncture Rig (TA-65). Approximately 185 g of samples were used, and the test was conducted using the return to start test in Compression with the following settings: pre-test speed of 10 mm/s, test speed of 5 mm/s; and post-test speed 5 mm/s. The puncture rig tines traveled 90% through the container according to the procedure of Texture Technologies Corp [30], and the results of firmness were reported in Newtons.

On each strawberry fruit, external (on opposing shoulder) and internal (adjacent to the central cavity) color parameters were determined by reflectance using a HunterLab colorimeter (ColorFlex Spectrophotometer, Hunter Associates Laboratory, Inc., Reston, VA, USA). The colorimeter was calibrated using a white tile with D65/10° Illuminant/observer and 8 mm aperture size, to estimate strawberries color space: Lightness (L*), Chroma (Chroma = [(a*)2 + (b*)2]1/2), and Hue angle (Hue = tan1 [b*/a*]. The Lightness value determines whether a sample is bright (high L*) or dark (low L*). The Chroma value indicates color saturation or intensity, and Hue value was determined as a color wheel, with red-purple color at 0°, yellow color at 90°, bluish-green color at 180°, and blue color at 270° [31]. The external and internal color of each sample were determined at room temperature (~22 °C) at each harvested period using a total of 5 strawberries per treatment.

### 3.8. Quantification of Sugars and Organic Acids

The quantifications of sucrose/D-glucose/D-fructose, citric acid, and L-malic acid were determined according to the procedure of Schwieterman et al. [21] using biochemical kits (per manufacturer’s instructions). The absorbance of samples was measured at 365 nm using a Spectramax Max Plus spectrophotometer (Molecular Devices, Sunnyvale, CA, USA). The sugars and organic acid concentrations were reported as mg/100 g.

The Sweetness Index (SI) content and Total Sweetness Index (TSI) were calculated to determine the sweetness perception of fruits using the following formulas [27]:

SI = (1.00 × [glucose]) + (2.30 × [fructose]) + (1.35 × [sucrose])(1)

TSI = (1.00 × [sucrose]) + (0.76 × [glucose]) + 1.50 × [fructose])(2)

### 3.9. Statistical Analysis

Experimental data were analyzed using the PROC Mixed model procedure of SAS (version 9.4, SAS Institute Inc., Cary, NC, USA). Analysis of variance (ANOVA) was performed with biopesticide treatment, harvest date, and biopesticide treatment × harvest date interaction. The data were analyzed separately by harvest day because the two-way interaction was significant (*p* ˂ 0.05). Thus, the effect of biopesticides treatments on the physicochemical characteristics of strawberry fruit was investigated by one-way analysis of variance using Tukey’s post hoc test. Conclusions were drawn at the 5% significance level (*p* < 0.05). Data are presented as the least squares means and their standard errors (SE). The physicochemical determinations in the samples were performed in triplicates.

## 4. Conclusions

This study demonstrated that the selected commercially available soil-applied biopesticides evaluated showed no negative effects on yield and fruit quality and thus could serve as alternatives to conventional products used. Further research and field trials are necessary to optimize the application of biopesticides for different strawberry (*Fragaria* × *ananassa*) varieties and production systems with regard to effects on yield, as well as fruit and nutritional quality postharvest.

## Figures and Tables

**Table 1:** Soil-applied biopesticides used, their active ingredients, application timing, and rates.

Treatment	Active Ingredient	Product Rate/ha (via Irrigation)	Application Timing
T1 (Untreated, UCtrl) ^†^	-	-	-
T2 (RootShield Plus + 3A) ^‡‡^	*Trichoderma harzianum* + *T. virens*	0.585 L/378.5 L	^††^ T + 22 wks + 16 wks
T3 (RootShield Plus + 6A)	*Trichoderma harzianum* + *T. virens*	0.585 L/378.5 L	T + 4 wks + 8 wks + 12 wks + 16 wks + 20 wks
T4 (Regalia + 6A)	Extract of *Reynoutria sachalinensis*	5.844 L	T + 4 wks + 8 wks + 12 wks + 16 wks + 20 wks
T5 (Double Nickel + 6A)	*Bacillus amyloliquefaciens* strain D747	2.337 L	T + 4 wks + 8 wks + 12 wks + 16 wks + 20 wks
T6 (Actinovate + 6A)	*Streptomycens lydicus* WYEC 108	0.263 L	T + 4 wks + 8 wks + 12 wks + 16 wks + 20 wks
T7 (Ridomil Gold + Abound FL + 3A, +Ctrl) ^‡^	Azoxystrobin + Mefenoxam	1.168 L	T + 8 wks + 16 wks
T8 (Regalia + 3A)	Extract of *Reynoutria sachalinensis*	5.844 L	T + 8 wks + 16 wks
T9 (Double Nickel + 3A)	*Bacillus amyloliquefaciens* strain D747	2.337 L	T + 8 wks + 16 wks
T10 (Actinovate + 3A)	*Streptomycens lydicus* WYEC 108	0.263 L	T + 8 wks + 16 wks

^†^ UCtrl = untreated control; ^‡^ +Ctrl = conventional or positive control; ^††^ T = time of transplanting; wks = weeks; ^‡‡^ 3A = three applications; 6A = six applications.

**Table 2:** Marketable strawberry (*Fragaria × ananassa*) fruit yield for the soil-applied biopesticide treatments.

Treatment	Marketable Fruit Number/Plot	Marketable Fruit Weight/Plot (g)
T1 (UCtrl) ^†^	87.00 ^cd^	1802.30 ^bc^
T2 (RootShield Plus + 3A) ^‡‡^	82.00 ^d^	1602.50 ^c^
T3 (RootShield Plus + 6A)	116.70 ^a–d^	2506.10 ^ab^
T4 (Regalia + 6A)	97.00 ^cd^	2012.43 ^bc^
T5 (Double Nickel + 6A)	122.70 ^a–c^	2564.40 ^ab^
T6 (Actinovate + 6A)	99.00 ^b–d^	1889.83 ^bc^
T7 (Ridomil Gold + Abound FL + 3A, +Ctrl) ^‡^	148.33 ^a^	3061.60 ^a^
T8 (Regalia + 3A)	142.70 ^a^	2916.53 ^a^
T9 (Double Nickel + 3A)	150.33 ^a^	3118.03 ^a^
T10 (Actinovate + 3A)	135.33 ^ab^	2827.03 ^a^
*p*-value	0.0050	0.0022

^†^ UCtrl = untreated control; ^‡^ +Ctrl = conventional or positive control; ^‡‡^ 3A = three applications; 6A = six applications. Means in the same column within each application treatment with different lowercase superscripts are significantly different (*p* ˂ 0.05) according to Tukey’s test.

**Table 3:** Effect of soil-applied biopesticide treatments on fruit size and total soluble solids of fresh strawberries (*Fragaria* × *ananassa*).

	Fruit Length (mm)			Fruit Major Diameter (mm)			Total Soluble Solids (°Brix)		
Treatment	Mid-April	Late April	Mid-May	Mid-April	Late April	Mid-May	Mid-April	Late April	Mid-May
T1 (UCtrl) ^†^	41.6	35.8 ^b^	26.4	39.1	35.3	28.0	7.07 ^bc^	7.67 ^a^	8.50 ^a^
T2	40.8	36.4 ^b^	28.6	34.6	33.6	28.8	7.43 ^ab^	7.55 ^a^	7.50 ^ab^
T3	44.7	37.2 ^b^	32.0	38.3	33.5	33.0	8.08 ^a^	6.83 ^b^	6.04 ^b^
T4	40.6	39.1 ^ab^	32.2	32.9	34.9	31.8	7.60 ^ab^	6.83 ^b^	8.00 ^a^
T5	41.8	39.1 ^ab^	31.9	35.7	36.1	32.8	7.27 ^a–c^	6.77 ^b^	7.70 ^a^
T6	43.2	42.1 ^a^	31.6	38.2	35.3	31.3	7.50 ^ab^	7.27 ^ab^	8.00 ^a^
T7 (+Ctrl) ^‡^	41.7	38.3 ^ab^	31.2	35.8	34.7	30.1	7.47 ^ab^	6.80 ^b^	7.92 ^a^
T8	40.2	38.8 ^ab^	31.7	34.7	35.2	30.9	6.32 ^c^	6.93 ^b^	7.92 ^a^
T9	40.4	36.2 ^b^	32.0	36.0	34.9	31.7	7.87 ^ab^	6.93 ^b^	7.20 ^ab^
T10	41.5	36.6 ^b^	28.2	35.4	33.5	30.2	7.55 ^ab^	6.93 ^b^	8.30 ^a^
SE	1.40	0.93	1.38	1.44	1.11	1.28	0.214	0.115	0.312
*p*-value	0.410	0.0005	0.050	0.064	0.724	0.189	<0.0001	<0.0001	0.0004

^†^ UCtrl = untreated control; ^‡^ +Ctrl = conventional or positive control. Means in the same column within each application treatment with different lowercase superscripts are significantly different (*p* ˂ 0.05) according to Tukey’s test.

**Table 4:** Effect of soil-applied biopesticide treatments on pH, titratable acidity, and total soluble solids/titratable acidity ratio of fresh strawberries (*Fragaria* × *ananassa*).

	pH			TA (%)			TSS/TA		
Treatment	Mid-April	Late April	Mid-May	Mid-April	Late April	Mid-May	Mid-April	Late April	Mid-May
T1 (UCtrl) ^†^	3.35 ^c–e^	3.36 ^d^	3.79 ^ab^	0.65 ^c^	0.83 ^a^	0.88	10.95 ^ab^	9.34 ^cd^	9.89 ^b^
T2	3.37 ^cd^	3.74 ^ab^	3.55 ^a–c^	0.67 ^c^	0.88 ^a^	0.80	11.15 ^a^	8.71 ^d^	9.85 ^b^
T3	3.39 ^bc^	3.75 ^ab^	3.69 ^a–c^	0.79 ^a^	0.62 ^b^	1.10	10.21 ^ab^	11.03 ^a–c^	9.59 ^b^
T4	3.33 ^c–e^	3.83 ^a^	3.72 ^a–c^	0.71 ^b^	0.65 ^b^	0.70	10.78 ^ab^	10.56 ^bc^	11.50 ^a^
T5	3.30 ^de^	3.68 ^bc^	3.85 ^a^	0.72 ^a–c^	0.61 ^b^	0.64	10.16 ^ab^	11.11 ^a–c^	12.07 ^a^
T6	3.50 ^a^	3.70 ^ab^	3.54 ^a–c^	0.68 ^c^	0.58 ^b^	1.10	10.95 ^ab^	12.68 ^a^	12.35 ^a^
T7 (+Ctrl) ^‡^	3.51 ^a^	3.66 ^bc^	3.53 ^a–c^	0.77 ^ab^	0.59 ^b^	0.75	9.61 ^b^	11.52 ^ab^	10.72 ^ab^
T8	3.46 ^ab^	3.71 ^ab^	3.42 ^bc^	0.65 ^c^	0.60 ^b^	1.21	9.64 ^b^	11.58 ^ab^	10.04 ^ab^
T9	3.34 ^c–e^	3.69 ^a–c^	3.52 ^a–c^	0.72 ^ab^	0.60 ^b^	0.80	11.00 ^ab^	11.72 ^ab^	8.95 ^b^
T10	3.27 ^e^	3.55 ^bc^	3.34^c^	0.66 ^c^	0.61 ^b^	1.24	11.47 ^a^	11.49 ^ab^	8.94 ^b^
SE	0.018	0.032	0.091	0.018	0.027	0.258	0.319	0.385	0.748
*p*-value	<0.0001	<0.0001	0.004	<0.0001	<0.0001	0.686	0.001	<0.0001	0.014

^†^ UCtrl = untreated control; ^‡^ +Ctrl = conventional or positive control; TA = titratable acidity (% as citric acid equivalent); TSS = total soluble solids (Brix). Means in the same column within each application treatment with different lowercase superscripts are significantly different (*p* ˂ 0.05) according to Tukey’s test.

**Table 5:** Effect of soil-applied biopesticide treatments on sugar content of fresh strawberries (*Fragaria* × *ananassa*).

	Glucose (mg/100 g)			Fructose (mg/100 g)			Sucrose (mg/100 g)		
Treatment	Mid-April	Late April	Mid-May	Mid-April	Late April	Mid-May	Mid-April	Late April	Mid-May
T1 (UCtrl) ^†^	1261 ^cd^	1993 ^ab^	2380 ^a^	1367 ^d^	2280	2801 ^a^	611	985	492
T2	1297 ^cd^	1980 ^ab^	2244 ^ab^	1534 ^cd^	2249	2681 ^ab^	709	1034	561
T3	1522 ^a–c^	2084 ^a^	1892 ^c^	1638 ^b–d^	2274	2347 ^b^	739	847	433
T4	1260 ^cd^	1887 ^b^	2099 ^a–c^	1471 ^d^	2316	2384 ^b^	630	798	404
T5	1192 ^d^	1892 ^b^	2022 ^bc^	1388 ^d^	2410	2509 ^ab^	541	896	384
T6	1223 ^d^	1991 ^ab^	1964 ^bc^	1403 ^d^	2483	2562 ^ab^	807	896	418
T7 (+Ctrl) ^‡^	1398 ^b–d^	2032 ^ab^	1898 ^c^	1602 ^b–d^	2368	2457 ^ab^	817	896	453
T8	1576 ^ab^	2099 ^a^	1949 ^b^	1877 ^a–c^	2238	2462 ^ab^	532	847	423
T9	1684 ^a^	2037 ^ab^	1897 ^c^	1941 ^ab^	2295	2478 ^ab^	601	925	433
T10	1617 ^ab^	1975 ^ab^	1918 ^b^	2087 ^a^	2306	2464 ^ab^	768	817	394
SE	52.87	36.90	66.30	78.78	61.38	74.47	92.02	79.01	61.29
*p*-value	<0.0001	0.008	0.0003	<0.0001	0.191	0.012	0.281	0.572	0.671

^†^ UCtrl = untreated control; ^‡^ +Ctrl = conventional or positive control. Means in the same column within each application treatment with different lowercase superscripts are significantly different (*p* ˂ 0.05) according to Tukey’s test.

**Table 6:** Effect of soil-applied biopesticide treatments on total sugar content of fresh strawberries (*Fragaria* × *ananassa*).

	Total Sugar (mg/100g)			Sweetness Index (mg/100 g)			Total Sweetness Index (mg/100g)		
Treatment	Mid-April	Late April	Mid-May	Mid-April	Late April	Mid-May	Mid-April	Late April	Mid-May
T1 (UCtrl) ^†^	3239 ^c–f^	5257	5674 ^a^	5229 ^d–f^	8565	9488 ^a^	3619 ^d–f^	5919	6503 ^a^
T2	3540 ^c–f^	5263	5487 ^ab^	5782 ^c–f^	8548	9169 ^ab^	3995 ^c–f^	5912	6292 ^ab^
T3	3899 ^a–d^	5205	4673 ^c^	6288 ^b–d^	8458	7876 ^c^	4353 ^b–d^	5842	5392 ^c^
T4	3361 ^c–f^	5000	4886 ^bc^	5495 ^c–f^	8291	8127 ^bc^	3795 ^d–f^	5706	5574 ^bc^
T5	3121 ^f^	5198	4915 ^bc^	5115 ^f^	8645	8312 ^a–c^	3529 ^f^	5949	5685 ^a–c^
T6	3434 ^c–f^	5370	4945 ^a–c^	5541 ^c–f^	8912	8421 ^a–c^	3842 ^c–f^	6134	5754 ^a–c^
T7 (+Ctrl) ^‡^	3808 ^b–e^	5296	4808 ^bc^	6176 ^b–e^	8689	8161 ^bc^	4276 ^b–e^	5993	5581 ^bc^
T8	3984 ^a–c^	5184	4835 ^bc^	6611 ^a–c^	8390	8184 ^bc^	4545 ^a–c^	5799	5598 ^bc^
T9	4226 ^ab^	5258	4809 ^bc^	6959 ^ab^	8567	8182 ^bc^	4792 ^ab^	5917	5592 ^bc^
T10	4472 ^a^	5098	4776 ^bc^	7453 ^a^	8382	8117 ^bc^	5127 ^a^	5777	5547 ^bc^
SE	121.21	87.03	146.72	210.90	168.96	246.25	143.88	114.24	168.64
*p*-value	<0.0001	0.240	0.001	<0.0001	0.391	0.003	<0.0001	0.390	0.003

^†^ UCtrl = untreated control; ^‡^ +Ctrl = conventional or positive control. Means in the same column within each application treatment with different lowercase superscripts are significantly different (*p* ˂ 0.05) according to Tukey’s test.

**Table 7:** Effect of soil-applied biopesticide treatments on organic acids content and firmness of fresh strawberries (*Fragaria* × *ananassa*).

	Citric Acid (%)			Malic Acid (%)			Firmness (g Force)		
Treatment	Mid-April	Late April	Mid-May	Mid-April	Late April	Mid-May	Mid-April	Late April	Mid-May
T1 (UCtrl) ^†^	0.71 ^a^	0.69 ^a^	0.70	0.13 ^a^	0.13	0.15 ^a^	4450 ^ab^	3307 ^a–c^	2832
T2	0.67 ^a^	0.65 ^a^	0.63	0.11 ^b^	0.13	0.15 ^a^	3556 ^b^	3628 ^a^	2302
T3	0.69 ^a^	0.62 ^ab^	0.61	0.11 ^b^	0.12	0.14 ^ab^	3958 ^b^	3391 ^a–c^	1951
T4	0.59 ^b^	0.56 ^bc^	0.64	0.14 ^a^	0.13	0.13 ^ab^	4388 ^ab^	3514 ^ab^	2526
T5	0.60 ^b^	0.56 ^bc^	0.57	0.14 ^a^	0.12	0.13 ^ab^	5441 ^a^	2562 ^b–d^	2545
T6	0.58 ^b^	0.57 ^bc^	0.58	0.11 ^b^	0.12	0.13 ^ab^	3979 ^b^	2729 ^a–d^	2142
T7 (+Ctrl) ^‡^	0.60 ^b^	0.52 ^b–d^	0.64	0.12 ^b^	0.13	0.13 ^ab^	4304 ^ab^	2263 ^d^	2249
T8	0.68 ^a^	0.45 ^d^	0.59	0.15 ^a^	0.11	0.12 ^ab^	3785 ^b^	2611 ^b–d^	2908
T9	0.71 ^a^	0.50 ^cd^	0.58	0.16 ^a^	0.11	0.11 ^b^	4408 ^ab^	2464 ^cd^	2637
T10	0.73 ^a^	0.53 ^cd^	0.60	0.16 ^a^	0.12	0.13 ^ab^	3895 ^b^	2705 ^a–d^	2882
SE	0.030	0.017	0.029	0.030	0.007	0.006	229.14	201.65	348.97
*p*-value	0.009	<0.0001	0.124	0.028	0.414	0.018	0.001	0.0005	0.550

^†^ UCtrl = untreated control; ^‡^ +Ctrl = conventional or positive control. Means in the same column within each application treatment with different lowercase superscripts are significantly different (*p* ˂ 0.05) according to Tukey’s test.

**Table 8:** Effect of soil-applied biopesticide treatments on internal color attributes of fresh strawberries (*Fragaria* × *ananassa*).

	Lightness_i_			Chroma_i_			Hue_i_		
Treatment	Mid-April	Late April	Mid-May	Mid-April	Late April	Mid-May	Mid-April	Late April	Mid-May
T1 (UCtrl) ^†^	48.00 ^ab^	42.14 ^a^	38.37 ^ab^	37.68 ^a–c^	39.86 ^a–c^	33.25 ^ab^	54.71 ^ab^	55.99 ^b^	56.86 ^cd^
T2	45.24 ^b^	40.47 ^ab^	41.35 ^a^	39.60 ^a^	39.63 ^a–c^	34.54 ^ab^	55.16 ^a^	56.55 ^ab^	55.92 ^d^
T3	45.84 ^b^	41.14 ^ab^	36.25 ^b^	38.12 ^ab^	41.32 ^ab^	36.22 ^a^	56.03 ^a^	56.48 ^ab^	57.00 ^b–d^
T4	45.34 ^b^	39.09 ^a–c^	39.09 ^ab^	35.56 ^bc^	41.44 ^ab^	33.95 ^ab^	55.01 ^a^	56.63 ^ab^	58.86 ^a–c^
T5	45.50 ^b^	36.36 ^c^	41.04 ^a^	36.72 ^a–c^	41.06 ^a–c^	34.44 ^ab^	55.82 ^a^	57.62 ^a^	57.18 ^b–d^
T6	45.20 ^b^	40.79 ^ab^	36.44 ^b^	35.62 ^bc^	37.66 ^c^	31.66 ^b^	56.93 ^a^	56.10 ^ab^	59.96 ^a^
T7 (+Ctrl) ^‡^	46.01 ^ab^	37.43 ^bc^	38.45 ^ab^	36.69 ^a–c^	42.12 ^a^	33.65 ^ab^	56.98 ^a^	55.73 ^b^	58.44 ^a–c^
T8	50.55 ^a^	39.44 ^a–c^	37.72 ^ab^	35.25 ^bc^	38.58 ^a–c^	33.11 ^ab^	52.31 ^b^	56.09 ^ab^	58.99 ^ab^
T9	47.32 ^ab^	37.41 ^bc^	39.37 ^ab^	35.11 ^c^	38.74 ^a–c^	34.24 ^ab^	55.34 ^a^	56.81 ^ab^	57.30 ^b–d^
T10	44.47 ^b^	40.46 ^ab^	39.87 ^ab^	36.99 ^a–c^	38.51 ^bc^	32.16 ^b^	56.48 ^a^	57.16 ^ab^	57.16 ^b–d^
SE	1.022	0.881	0.850	0.642	0.782	0.833	0.541	0.344	0.458
*p*-value	0.0016	˂0.0001	0.0001	˂0.0001	˂0.0002	0.0140	˂0.0001	˂0.0047	˂0.0001

^†^ UCtrl = untreated control; ^‡^ +Ctrl = conventional or positive control. Means in the same column within each application treatment with different lowercase superscripts are significantly different (*p* ˂ 0.05) according to Tukey’s test. Lightness_i_, L*, internal; Chroma_i_ = [(a*)^2^ + (b*)^2^]^1/2^, internal; Hue_i_: hue angle = tan^−1^ [b*/a*], internal.

**Table 9:** Effect of soil-applied biopesticide treatments on external color attributes of fresh strawberries (*Fragaria* × *ananassa*).

	Lightness_e_			Chroma_e_			Hue_e_		
Treatment	Mid-April	Late April	Mid-May	Mid-April	Late April	Mid-May	Mid-April	Late April	Mid-May
T1 (UCtrl) ^†^	24.09 ^a^	21.56	21.37 ^a–c^	19.66 ^b–d^	20.67 ^a^	20.06 ^ab^	69.90 ^bc^	68.01 ^b^	67.66
T2	23.85 ^a^	21.29	19.95 ^b^	18.94 ^cd^	17.51 ^b^	20.69 ^ab^	70.63 ^b^	71.12 ^ab^	65.55
T3	21.98 ^c^	21.82	19.93 ^b^	17.32 ^d^	18.51 ^ab^	19.02 ^b^	73.66 ^a^	71.30 ^ab^	69.17
T4	21.79 ^c^	21.51	23.31 ^a^	21.63 ^a–c^	17.10 ^b^	20.48 ^ab^	67.78 ^cd^	72.96 ^a^	69.21
T5	22.69 ^a–c^	21.47	22.20 ^ab^	23.19 ^a^	16.27 ^b^	21.64 ^ab^	66.82 ^d^	72.61 ^a^	68.39
T6	22.21 ^bc^	21.09	20.55 ^bc^	19.12 ^cd^	16.13 ^b^	20.03 ^ab^	70.33 ^bc^	74.11 ^a^	69.19
T7 (+Ctrl) ^‡^	23.53 ^ab^	21.47	22.15 ^ab^	22.10 ^ab^	17.33 ^b^	22.79 ^a^	68.04 ^b–d^	71.19 ^ab^	67.54
T8	22.75 ^a–c^	21.18	19.83 ^c^	20.51 ^a–c^	16.20 ^b^	18.76 ^b^	69.34 ^b–d^	73.65 ^a^	69.70
T9	22.05 ^bc^	21.05	22.52 ^ab^	19.36 ^b–d^	16.44 ^b^	21.49 ^ab^	69.73 ^bc^	73.22 ^a^	67.65
T10	23.05 ^a–c^	21.94	22.08 ^ab^	20.59 ^a–c^	18.07 ^ab^	21.84 ^ab^	69.88 ^bc^	72.74 ^a^	69.78
SE	0.331	0.239	0.454	0.611	0.539	0.704	0.607	0.822	1.025
*p*-value	˂0.0001	0.125	˂0.0001	˂0.0001	˂0.0001	0.0008	˂0.0001	˂0.0001	0.120

^†^ UCtrl = untreated control; ^‡^ +Ctrl = conventional or positive control; SE = standard error. Means in the same column within each application treatment with different lowercase superscripts are significantly different (*p* ˂ 0.05) according to Tukey’s test. Lightness_e_: L*, external; Chroma_e_ = [(a*)^2^ + (b*)^2^]^1/2^, external; Hue_e_: Hue angle = tan^−1^ [b*/a*], external.

**Table 10:** Precipitation amounts, days of precipitation, temperature, relative humidity, and wind speed during the strawberry (*Fragaria* × *ananassa*) growing season from October 2018 to May 2019.

Date	Precipitation (mm)	Days of Precipitation	Temperature Min ± Max (°C)	Relative Humidity (%)	Wind Speed (mph)
1–31 October 2018	14.98	5	16.6 ± 26.6	47.87	5.71
1–30 November 2018	61.21	10	8.1 ± 18.6	48.83	6.14
1–31 December 2018	167.38	10	7.3 ± 17.2	48.90	6.81
1–31 January 2019	128.77	15	4.8 ± 15.9	49.77	6.22
1–28 February 2019	42.16	17	9.7 ± 18.1	49.89	6.86
1–31 March 2019	14.22	6	10.1 ± 21.2	50.64	7.03
1–30 April 2019	50.54	8	13.4 ± 25.3	49.60	7.33
1–31 May 2019	250.44	10	19.3 ± 27.9	57.16	7.51
Total	729.7	81			

## Data Availability

Data are contained within the article.
